# HPLC-UV and UPLC-MS/MS methods for the simultaneous analysis of sildenafil, vardenafil, and tadalafil and their counterfeits dapoxetine, paroxetine, citalopram, tramadol, and yohimbine in aphrodisiac products†

**DOI:** 10.1039/d0ra10324a

**Published:** 2021-02-18

**Authors:** Mohamed A. Abdelshakour, Randa A. Abdel Salam, Ghada M. Hadad, Dina M. Abo-ElMatty, Eman A. Abdel Hameed

**Affiliations:** Forensic Medicine Administration, Ministry of Justice Egypt; Department of Pharmaceutical Analytical Chemistry, Faculty of Pharmacy, Suez Canal University Ismailia Egypt; Department of Pharmaceutical Analytical Chemistry, Faculty of Pharmacy, Sinai University, Kantara Branch Egypt; Department of Biochemistry, Faculty of Pharmacy, Suez Canal University Ismailia Egypt; Department of Pharmaceutical Analytical Chemistry, Faculty of Pharmacy, Port Said University Egypt emanali_19@hotmail.com +20-64-3561877 +20-01224448268

## Abstract

In recent times, the counterfeiting of pharmaceuticals has been considered a serious trouble especially in developing countries that acquire poor inspection programs. Sildenafil, vardenafil and tadalafil (phosphodiesterase type 5 inhibitors) products have gained wide popularity in treating sexual disorders, for which they are subjected to counterfeiting. For this purpose, a simple, rapid, and novel HPLC method with ultraviolet detection has been simply developed for the simultaneous determination of vardenafil, sildenafil, and tadalafil, and their counterfeits (dapoxetine, paroxetine, citalopram, tramadol and yohimbine) in pharmaceutical dosage forms and counterfeit products such as instant coffee and honey. The separation was carried out on a C_18_ column, with acetonitrile and an aqueous 0.05% formic acid solution as the mobile phase with a gradient program and at a flow rate of 1 mL min^−1^. UV detection was accurately set at 230 nm. The total run time was 11 min for elution of these eight drugs. A UPLC-MS/MS method was also developed, by which compounds were separated in only 6 min, and it was used as a confirmatory tool for studied compounds by identification of their mass spectra. Proposed methods were validated by following ICH guidelines. Both methods were found to be linear, specific, precise and accurate, and they were efficiently applied to analyze 50 commercial products including honey sachets, instant coffee and pharmaceutical products marketed as aphrodisiacs and suspected to contain PDE5-inhibitors.

## Abstract 2

In recent times, the counterfeiting of pharmaceuticals has been considered a serious trouble especially in developing countries that acquire poor inspection programs.

## Graphical abstract

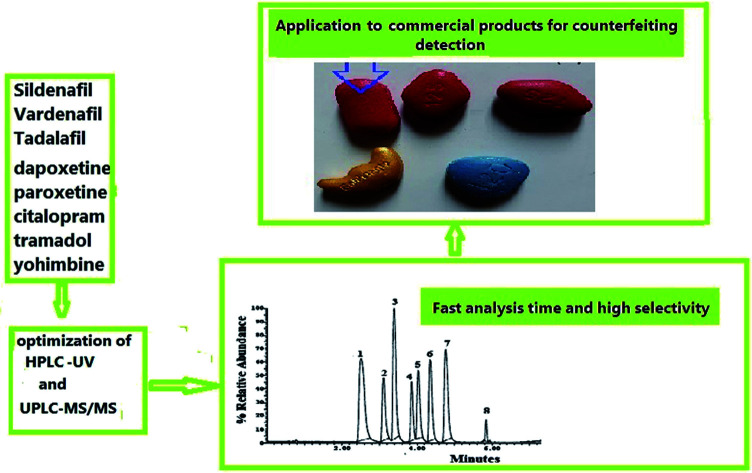

## 1. Introduction

Erectile dysfunction (ED) is really considered the most popular sexual dysfunction. ED is described as the lack of ability to have and/or keep an erection enough for a satisfying sexual act. ED is a serious medical problem that may affect the quality of life and cause anxiety, loss of self-confidence, and sadness. The mental stress because of ED could have many effects on the interaction of patients with others.^1^ Common causes for ED are diabetes, hypertension, cardiovascular disease, hyperlipidemia, injuries, obesity, anxiety, increased age, stress, smoking, drug use or alcohol use.^2^ Phosphodiesterase type 5 (PDE-5) inhibitors are broadly taken as a therapy of choice for those patients with ED who do not have a specific contraindication preventing their use.^3^ In Egypt, sildenafil (SLD), tadalafil (TAD), and vardenafil (VAR) are approved by the Egyptian Ministry of Health for clinical use. Another substance, which is used for the treatment of ED, is yohimbine (YHB). YHB is obtained from the bark and roots of an African yohimbe tree. It is a relatively selective alpha-2-adrenoceptor antagonist, causing raised cholinergic and reduced adrenergic tone. Its extract has long been used as an aphrodisiac and as a remedy for psychogenic erectile insufficiency.^4^ Premature ejaculation (PME) is another male sexual dysfunction. PME is defined as the inability to control or delay ejaculation, which results in dissatisfaction for the patient.^5^ Premature ejaculation is classified into four subtypes: lifelong PME, acquired PME, variable PME and subjective PME. There are six major types of treatment for PME: daily use of selective serotonin reuptake inhibitors (SSRIs), the use of dapoxetine (DPX) on-demand, clomipramine, topical local anesthetics, tramadol (TRM) or PDE-5 inhibitors. Only DPX 30 and 60 mg has been registered by European Union agency (EMA) as a rapid-acting SSRI for the treatment of PME on-demand, while other treatments are considered off-label.^6^ Moreover, some studies suggest that the SSRI paroxetine (PRX) may be administered to treat premature ejaculation.^7^ Citalopram (CTP) is another SSRI recommended by some studies for the treatment of premature ejaculation.^8^ Due to the popularity, PDE-5 inhibitors (SLD, VAR and TAD) and products containing these compounds are often subjected to counterfeiting. Moreover, herbal products and dietary supplements adulterated with these compounds have been found in the market.^9–17^

Some clinical trials suggested the use of a combination of SLD with DPX, SLD with PRX,^18,19^ and SLD with TRM^20^ for the treatment of PME. In addition, a report in 2010 revealed that counterfeit DPX sold on-line contains unrevealed SLD.^21^ Some studies showed that SLD and its analogues were found as adulterants in an herbal supplement.^22–24^ In 2018, SLD and TRM were found in a product sold in herb outlets, in Iran (Tehran).^25^ Therefore, there was a real need for paying more attention for the analysis of counterfeit pharmaceutical products and herbal and food supplements used for the treatment of sexual dysfunction.

The literature declares that many analytical approaches were reported for simultaneous quantification of PDE-5 inhibitors SLD, TAD, and VAR in counterfeit drugs, pharmaceutical products and dietary supplements using different analytical techniques including TLC and HPLC-PDA-MS methods,^13^ HPLC-UV-ESI-MS,^9^ LC-MS,^26^ LC-MS/MS,^27–29^ LC/HRMS,^30^ HPLC-DAD and LC-MS/MS.^10^ SLD, TAD and YHB was analyzed by pulsed amperometric detection using a gold electrode coupled to HPLC separation.^31^ SLD, TAD, VAR and YHB were determined by LC-MS/MS^12,14^ and LC-diode array detector-quadrupole-time-of-flight (DAD-QTOF) system.^16^ TRM, SLD, and TAD were determined by HPLC using a calixarene stationary phase,^32^ TRM, SLD, DPX, and YHB were also determined with other compounds by HPLC-UV.^33^ One article was reported for the determination of SSRIs (DPX and PRX) with the three PDE-5 inhibitors (SLD, TAD, and VAR) by HPLC-DAD.^34^

The goal of this study was to develop simple and accurate analytical methods for simultaneous quantification of PDE-5 inhibitors (SLD, TAD, and VAR), YHB, TRM and common SSRIs (CTP, PRX and DPX), as this mixture was not separated previously followed by applying them to analyze counterfeited products widely used in the Egyptian market for treatment of male sexual dysfunctions.

## 2. Experimental

### 2.1. Instrumentation

#### 2.1.1. HPLC-UV analysis

An HPLC (Shimadzu, Kyoto, Japan) instrument was equipped with a model series LC-10 ADVP pump, SCL-10 AVP system controller, DGU-12 A Degasser, Rheodyne 7725i injector with 5 or 20 μL loop and a SPD-10AVP UV-VIS detector. An HPLC column oven, DALIAN REPLETE®, Hong Kong, was used. Data acquisition was performed using the Class-VP software.

#### 2.1.2. UPLC-MS/MS analysis

A Waters Acquity TM (USA) UPLC-system was equipped with a quaternary pump, autosampler. The tandem mass spectrometer was operated using the Waters Aquity TM TQD (triple quad detector) MS/MS using multiple reactions monitoring (MRM). An electrospray ionization (ESI) interface in the positive ionization mode was used. Mass spectrometer parameters were set on the positive mode, source temperature 150 °C, cone voltage 30 eV, capillary voltage 3 kV, desolvation temperature 440 °C, cone gas flow rate 50 L h^−1^, and desolvation gas flow rate 900 L h^−1^. Mass spectra were detected in the ESI between *m*/*z* 100–1000. Data acquisition and data integration were done using the MassLynx 4.1 SCN805 Software solution.

### 2.2. Materials and reagents

Pharmaceutical-grade authentic standards of SLD, TAD, VAR, DPX, PRX, CTP, YHB and TRM were used and verified to acquire a purity of 99.6 : 99.9% (w/w), on dry weight basis. Methanol and acetonitrile were of HPLC grade (BDH, Poole, UK) and formic acid used was of high analytical grade. Suspected counterfeit samples were obtained from local markets and pharmacies or purchased online.

### 2.3. Standard solutions

Stock solutions of the studied analytes were prepared by dissolving SLD, TAD, VAR, DPX, PRX, CTP, YHB and TRM separately in methanol to obtain a concentration of 0.5 mg mL^−1^ and stored at 4 °C till preparation of working solutions.

The working solutions were prepared by further dilution of the stock solutions with a specified mobile phase ratio of 0.05% formic acid in water : acetonitrile (50 : 50) to reach the concentration range stated for HPLC and UPLC-MS/MS methods.

### 2.4. Sample preparation

#### 2.4.1. Tablets and capsules

Ten tablets or capsules of each dosage form were separately weighed and finely powdered. Then, 10% of the tablet weight or capsule content was then accurately weighed, taken separately in 25 mL volumetric flasks, dissolved in 15 mL of methanol using an ultrasonic bath (5 min) and cooled to room temperature. The solutions were diluted to the required volume with the same solvent and then filtered using a 0.45 μm membrane filter (Millipore, Milford, MA) in case of the HPLC method or a 0.2 μm membrane filter (Millipore, Milford, MA) in case of the UPLC-MS/MS method. The first filtrates were removed and the rest were used as stock sample solutions. Further dilution was made with the mobile phase (0.05% formic acid in water : acetonitrile (50 : 50)) by taking 0.2 mL of the filtered stock sample solution and making up the volume to 10 mL. The general procedures for the HPLC-UV and UPLC-MS/MS approaches explained were followed, and the concentrations of the found drugs were calculated.

#### 2.4.2. Instant coffee

The contents of three sachets of each brand were mixed and powdered. A quantity of the powder of each brand equivalent to 10% of the sachet weight was then mixed with 10 mL methanol, sonicated for 20 min, and then centrifuged. The supernatants were diluted to the required volume with the same solvent and then filtered through membrane filters. The first filtrates were removed and the rest were used as stock sample solutions. Further dilution was made with the mobile phase (0.05% formic acid in water : acetonitrile (50 : 50)) by taking 0.2 mL of the filtered stock sample solution and making up to 10 mL. The general procedures for the HPLC-UV and UPLC-MS/MS approaches explained were followed, and the concentrations of the found drugs were calculated.

#### 2.4.3. Honey

One gram of each sample was simply dissolved in 10 mL methanol, sonicated for 20 min, cooled to room temperature and then centrifuged and filtered through membrane filters. The first filtrates were removed and the rest were used as stock sample solutions. Further dilution was made with the mobile phase (0.05% formic acid in water : acetonitrile (50 : 50)) by taking 0.2 mL of the filtered stock sample solution and making up to 10 mL. The general procedures for the HPLC-UV and UPLC-MS/MS approaches explained were followed, and the concentrations of the found drugs were calculated.

### 2.5. Samples analysis

The developed approaches were efficiently applied for the analysis of 50 different samples labeled to contain SLD, VAR, YHB, and TAD and (or) labeled as totally natural herbal ingredients, traded in the Egyptian market.

### 2.6. Chromatographic conditions

#### 2.6.1. HPLC-UV analysis

HPLC separation and quantitation were performed using a Phenomenex® (5 μm particle size) C_18_ column (150 × 4.6 mm (i.d.)). The mobile phase was water containing 0.05% formic acid (aqueous phase A) and acetonitrile (organic phase B). The gradient program was constructed as follows: 0–2 min 20% A, 2–7 min gradient up to 30% B, 7–8 min gradient up to 40% B, 8–10 min gradient up to 50% B and 10–12 min gradient up to 60% B, and 12–14 min gradient to 40% B and 14–15 min gradient to 20% B. The flow rate was set to be 1 mL min^−1^. Quantitation was performed with UV detection at 230 nm. All determinations were carried out at 25 °C. The volume injected was 5 μL.

#### 2.6.2. UPLC-MS/MS analysis

First, 1 μL of samples were injected into the UPLC comprising a C_18_ column (ACQUITY UPLC-BEH C18 1.7 μm particle size (2.1 × 50 mm) column) kept at 25 °C and a flow rate of 0.2 mL min^−1^. The separation of studied compounds was achieved by gradient elution: aqueous 0.1% formic acid as aqueous phase A and acetonitrile as organic phase B. The gradient program was built as follows: 0–1 min 20% B, 1–2 min gradient up to 30% B, 2–4 min gradient up to 40% B and 4–6 min gradient up to 50% B. After 6 min, the gradient was returned to the initial condition. A UPLC-MS/MS detector was set at the multiple reaction monitoring mode (MRM), to monitor the transition of molecular ions to the product ions with the aid of an electrospray ionization positive ion mode (ESI^+^) used for the monitoring of transition pairs of the studied analytes.

## 3. Results and discussion

There is no doubt that the spread of social media and the increase in the number of satellite channels, and its connection to every home, have provided platforms for drug counterfeiters and promoters to promote their products of counterfeit drugs. Especially, aphrodisiac drugs often deceive some people with slogans of “natural products, have no side effects and suitable for heart patients and diabetics” and claim that their products treat erectile dysfunction and premature ejaculation. Following the success of inspectors in seizing many of the counterfeits and non-registered drugs in the Egyptian market, there was an urgent need to find a method for the concurrent determination of the most common PDE-5 inhibitors (SLD, VAR, and TAD), and other drugs that are commonly used to manage premature ejaculation (YHB, CTP, PRX, DPX and TRM).

### 3.1. Optimization of chromatographic conditions

It is very important to consider the drugs' acid–base properties first, and hence, the p*K*_a_ values of the studied drugs were determined: TRM (basic p*K*_a_ = 9.41, acidic p*K*_a_ = 13.8), YHB (p*K*_a_ = 7.65, acidic p*K*_a_ = 14.68), VAR (basic p*K*_a_ = 6.2, acidic p*K*_a_ = 8.01), SLD (basic p*K*_a_ = 5.99, acidic p*K*_a_ = 11.14), CTP (p*K*_a_ = 9.78), PRX (p*K*_a_ = 9.77), DPX (p*K*_a_ = 9.04) and TAD (basic p*K*_a_ = 4.2, acidic p*K*_a_ = 15.17),^35^ all of the studied compounds were basic except TAD. In case of ionisable analytes, the mobile phase pH can highly influence the analyte retention time in case of HPLC and sensitivity in case of LC/MS methods. In our study, the mobile phase pH should be acidic, to ionize these basic drugs and consequently decrease their retention times and also enhance their detection in the positive ion mode.

**Fig. 1:**
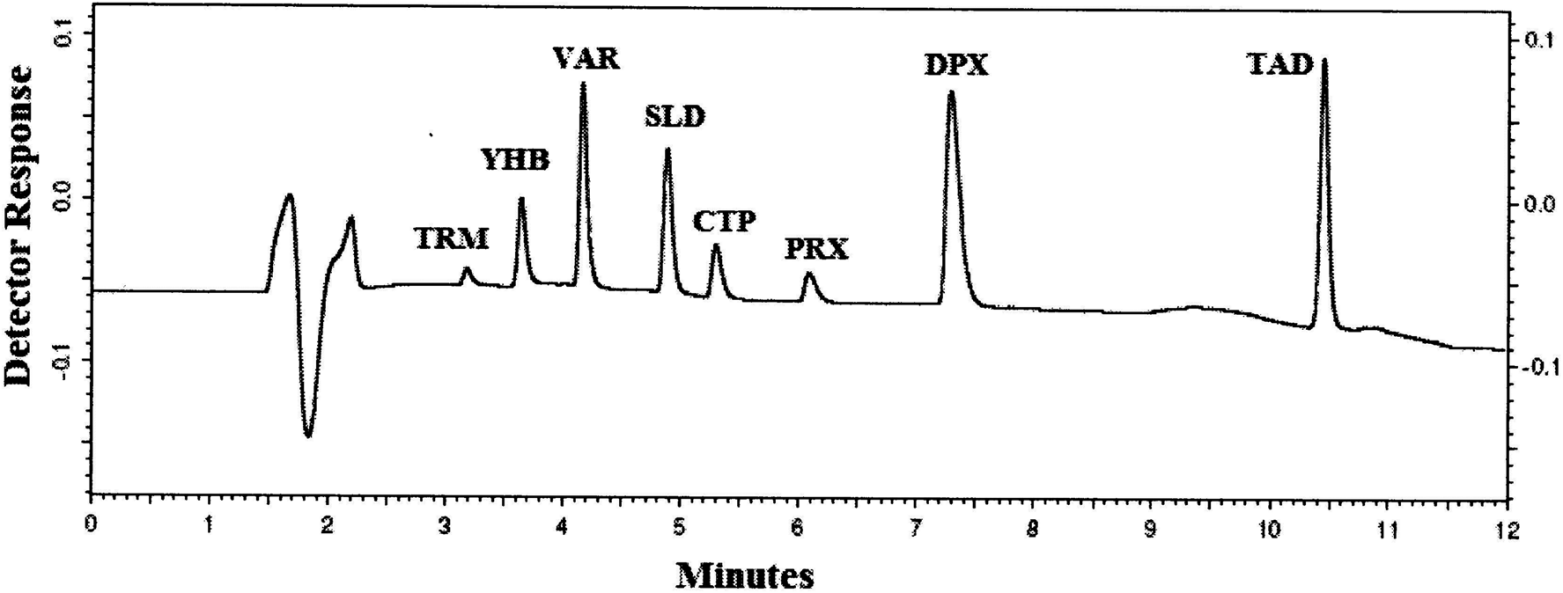
HPLC-UV chromatogram of 5 μL injection of standard prepared mixture containing 10 μg mL^−1^ of TRM, YHB, VAR, SLD, CTP, PRX, DPX and TAD.

Optimization of the chromatographic condition was difficult, and many columns were examined: C_8_, CN, and C_18_ columns. Upon trying C_8_ and CN columns that are more polar stationary phases than C_18_ and due to the polarity of these studied compounds at acidic pH, DPX and PRX were highly tailed, while TAD was retained on both columns. Therefore, using the C_18_ column was crucial for such separation offering good resolution for all the studied drugs, as indicated in Table 1. Different column temperatures were examined (25 °C, 30 °C and 35 °C), and it was found that by rising the temperature of the column to 35 °C, all obtained peaks became sharp (Fig. 1). Different mobile phase combinations were tried (acetonitrile with ammonium acetate or ammonium formate buffer, methanol with acetate or formate buffer, or mixture of acetonitrile and methanol as the organic phase with ammonium acetate or ammonium formate), and all these trails gave bad separation even after changing the buffer pH in the range from 3.5 to 5.0. Replacing the buffer with formic acid gave better separation for the critical pairs TRM with YHB and SLD with CTP. Gradient elution is used to simultaneously analyze these eight drugs because of their different structures and physicochemical properties; it helped to push strongly retained compounds, and consequently, shorten the analysis time, improve the quality of separation, and diminish peak tailing. Several time programs with different percentages of acetonitrile were tested to enhance the resolution of TRM with YHB and SLD with CTP and also to decrease the retaining of DPX and TAD on the analytical column. Increasing acetonitrile concentration to more than 30% in the first 5 minutes led to inadequate separation and overlap of TRM, YHB, SLD and CTP peaks. At a lower acetonitrile concentration (<40%) after 8 minutes, separation occurred but excessive tailing for DPX and TAD was observed. For wavelength selection, the spectra of the studied drugs were tested and the maximum wavelengths were noticed at 215 nm, 220 nm, 260 nm, 229 nm, 227 nm, 280 nm, 230 nm and 280 nm for TRM, YHB, VAR, SLD, CTP, PRX, DPX and TAD, respectively. Based on the spectra of the studied compounds, two wavelengths were examined, namely, 214 nm and 230, of which 230 nm showed more selectivity for the studied compounds with minimal noise than the shorter wavelength 214 nm. The separation was satisfactory when 0.05% formic acid in water was used as aqueous phase A and acetonitrile as organic phase B by gradient elution, as previously explained in Section 2.6, which gave very good resolution for the separation of TRM, YHB, VAR, SLD, CTP, PRX, DPX and TAD within 11 minutes (Fig. 1). The selectivity of the HPLC-UV method is demonstrated in Table 1.

The system suitability test results of TRM, YHB, VAR, SLD, CTP, PRX, DPX and TAD using HPLC-UV method and MS/MS transitions, retention times for the selected compounds using UPLC-MS/MS method

**Table:** 

HPLC-UV					
Compound	Retention time^a^ (min)	Capacity factor *k*	Selectivity factor^b^*α*	Resolution^b^*R*_s_	Tailing factor
TRM	3.20	3.57	1.16 (a_1_)	5.30 (b_1_)	1.03
YHB	3.60	4.14	1.21 (a_2_)	6.10 (b_2_)	0.96
VAR	4.20	5.00	1.21 (a_3_)	7.40 (b_3_)	0.98
SLD	4.94	6.06	1.10 (a_4_)	4.90 (b_4_)	0.96
CTP	5.35	6.64	1.17 (a_5_)	8.90 (b_5_)	1.04
PRX	6.14	7.77	1.22 (a_6_)	10.80 (b_6_)	1.02
DPX	7.36	9.51	1.46 (a_7_)	15.30 (b_7_)	1.10
TAD	10.40	13.86			1.09

a The retention time of unretained peak is 0.70 min.

b a_1_, b_1_ are *α* and *R*_s_ calculated for TRM, and YHB. a_2_, b_2_ are *α* and *R*_s_ calculated for YHB and VAR. a_3_, b_3_ are *α* and *R*_s_ calculated for VAR, and SLD. a_4_, b_4_ are *α* and *R*_s_ calculated for SLD, and CTP. a_5_, b_5_ are *α* and *R*_s_ calculated for CTP, and PRX. a_6_, b_6_ are *α* and *R*_s_ calculated for PRX, and DPX. a_7_, b_7_ are *α* and *R*_s_ calculated for DPX, and TAD.

**Table:** 

UPLC-MS/MS			
Compound	Retention time (min)	Precursor ion [M + H]^+^ (*m*/*z*)	Fragment ions (*m*/*z*)
TRM	2.62	264.2	265.3
YHB	3.15	355.3	356.3–357.3
VAR	3.45	489.3	490.3
SLD	3.90	475.3	476.3–477.3
CTP	4.12	325.3	326.3–327.3
PRX	4.41	330.2	331.2–332.3
DPX	4.82	306.3	307.3–308.3
TAD	5.81	390.2	391.2

**Fig. 2:**
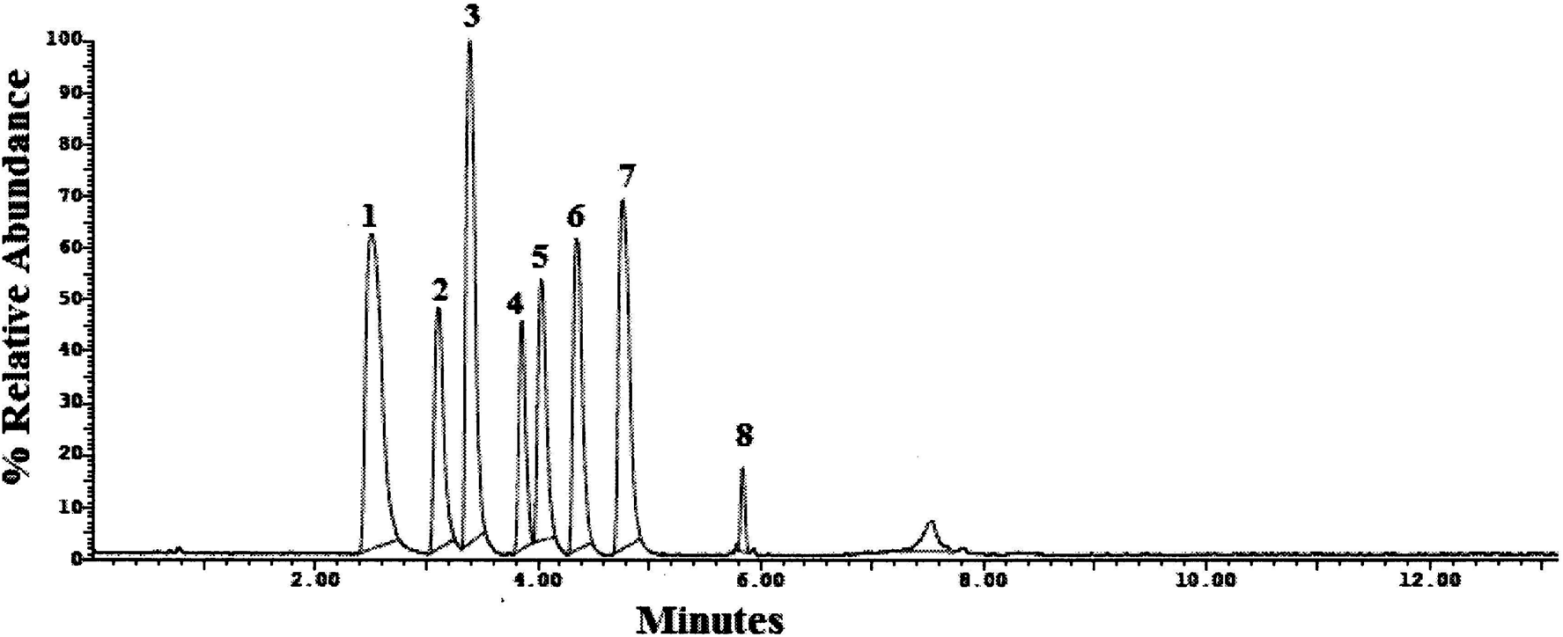
UPLC-MS/MS chromatogram of standard prepared mixture containing (1) TRM, (2) YHB, (3) VAR, (4) SLD, (5) CTP, (6) PRX, (7) DPX and (8) TAD.

**Fig. 3:**
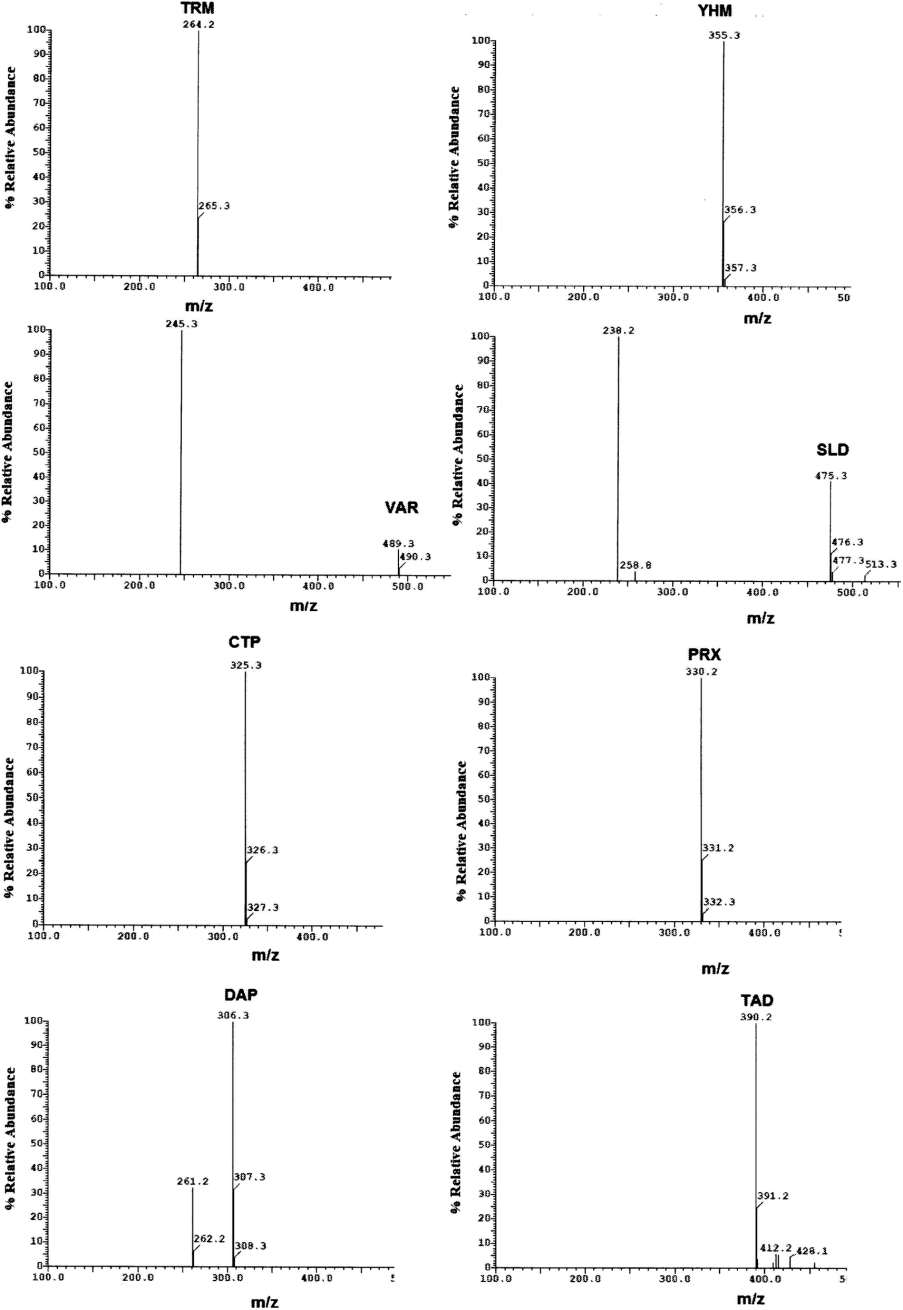
Mass spectra of standard prepared mixture containing TRM, YHB, VAR, SLD, CTP, PRX, DPX and TAD.

Method transfer to UPLC-MS/MS was easy and optimization was facilitated due to the pre-studied and validated HPLC-UV method, however with little tuning of the separation conditions to fit the specifications of UPLC-MS/MS instrument. Separation was achieved by gradient elution of aqueous 0.1% formic acid as aqueous phase A and acetonitrile as organic phase B. The gradient program was previously explained in Section 2.6. The standard for each analyte was auto-tuned in a positive mode separately according to its masses, namely, 263.200, 354.300, 488.300, 474.300, 324.300, 329.200, 305.300 and 389.200 for TRM, YHB, VAR, SLD, CTP, PRX, DPX and TAD, respectively. The optimum separation for each analyte is presented in Fig. 2. The positive ion mode was selected for MRM analysis, where the protonated precursor ions [M + H]^+^ of TRM, YHB, VAR, SLD, CTP, PRX, DPX and TAD in the Q1 full-scan mass spectrum were predominant at *m*/*z* values (listed in Table 1). The fragmentation pattern obtained in the mass spectra was used for the prediction and identification of studied compounds (Fig. 3).

### 3.2. Validation of the methods

#### 3.2.1. Linearity

The linearity of the proposed methods was assessed by analyzing different concentrations of each drug. Seven concentrations were selected, namely, 0.1, 0.2, 0.5, 1, 5, 15, and 30.0 μg mL^−1^ for TRM, YHB, VAR, SLD, CTP, PRX, DPX and TAD in the HPLC-UV method, while in UPLC-MS/MS, 10, 20, 25, 50, 70, 90, and 100 ng mL^−1^ were used for the studied compounds. Each concentration level was injected 3 times in order to provide information on the variation in peak area values between samples of the same concentration. The linearity of the calibration graphs was assessed by the high value of the determination coefficient *r*^2^ (Table 2). The parameters for the regression equations of the methods obtained by least-squares treatment of the results are given in Table 2.

**Table:** Analytical parameters for the analysis of TRM, YHB, VAR, SLD, CTP, PRX, DPX and TAD by the proposed HPLC-UV and UPLC-MS/MS analytical methods

Parameter	TRM	YHB	VAR	SLD	CTP	PRX	DPX	TAD
**HPLC-UV**								
Linearity range (μg mL^−1^)	0.1–30	0.1–30	0.1–30	0.1–30	0.1–30	0.1–30	0.1–30	0.1–30
Determination coefficient (*r*^2^)	0.99996	0.99997	0.99996	0.99996	0.99988	0.99989	0.99998	0.99997
LOD (μg mL^−1^)	0.0093	0.0092	0.0130	0.0055	0.0293	0.0280	0.0251	0.0071
LOQ (μg mL^−1^)	0.031	0.030	0.042	0.018	0.098	0.093	0.084	0.024
Regression equation(*y*)^a^: slope (*b*)	2.70 × 10^5^	2.10 × 10^5^	4.93 × 10^5^	4.33 × 10^5^	3.29 × 10^5^	1.16 × 10^4^	2.52 × 10^5^	4.41 × 10^5^
Standard deviation of the slope (*s*^b^)	1.19 × 10^3^	2.79 × 10^3^	2.63 × 10^3^	1.11.x10^3^	4.12 × 10^3^	1.38 × 10^2^	2.70 × 10^3^	1.32 × 10^3^
Confidence limit of the slope^b^	2.695 × 10^5^ to 2.700 × 10^5^	2.089 × 10^5^ to 2.110 × 10^5^	4.920 × 10^5^ to 4.940 × 10^5^	4.325 × 10^5^ to 4.334 × 10^5^	3.274 × 10^5^ to 3.306 × 10^5^	1.154 × 10^4^ to 1.165 × 10^4^	2.509 × 10^5^ to 2.530 × 10^5^	4.405 × 10^5^ to 4.415 × 10^5^
Relative standard deviation of the slope (%)	0.44	0.39	0.53	0.25	1.25	1.19	1.07	0.301
Intercept (*a*)	6.03 × 10^4^	−5.62 × 10^3^	6.22 × 10^3^	4.51 × 10^3^	2.22 × 10^4^	−1.44 × 10^2^	−1.70 × 10^4^	9.11 × 10^3^
Standard deviation of the intercept (*s*^a^)	1.44 × 10^4^	3.38 × 10^4^	3.18 × 10^4^	1.35 × 10^4^	4.99 × 10^4^	1.68 × 10^3^	3.27 × 10^4^	1.61 × 10^4^
Confidence limit of the intercept	5.97 × 10^4^ to 6.08 × 10^4^	−5.74 × 10^3^ to −5.49 × 10^3^	6.09 × 10^3^ to 6.34 × 10^3^	4.45 × 10^3^ to 4.56 × 10^3^	2.03 × 10^4^ to 2.41 × 10^4^	−1.50 × 10^2^ to −1.37 × 10^2^	−1.82 × 10^4^ to −1.56 × 10^4^	9.04 × 10^3^ to 9.17 × 10^3^

**UPLC-MS/MS**								
Linearity range (ng mL^−1^)	10–100	10–100	10–100	10–100	10–100	10–100	10–100	10–100
Determination coefficient (*r*^2^)	0.99997	0.99979	0.99989	0.99997	0.99998	0.99979	0.99969	0.99996
LOD (ng mL^−1^)	0.008	0.042	0.033	0.011	0.011	0.041	0.044	0.027
LOQ (ng mL^−1^)	0.028	0.140	0.109	0.036	0.036	0.137	0.0147	0.091
Regression equation(*y*)^a^: slope (*b*)	1.96 × 10^5^	8.79 × 10^4^	1.73 × 10^5^	7.72 × 10^4^	7.05 × 10^4^	1.09 × 10^5^	2.47 × 10^5^	3.07 × 10^5^
Standard deviation of the slope (*s*^b^)	6.95 × 10^2^	1.58 × 10^3^	2.41 × 10^3^	3.63 × 10^2^	3.62 × 10^2^	1.91 × 10^3^	4.64 × 10^3^	3.59 × 10^3^
Confidence limit of the slope^b^	19.57 × 10^4^ to 19.62 × 10^4^	8.73 × 10^4^ to 8.84 × 10^4^	17.20 × 10^4^ to 17.39 × 10^4^	7.70 × 10^4^ to 7.73 × 10^4^	7.03 × 10^4^ to 7.06 × 10^4^	10.82 × 10^4^ to 10.97 × 10^4^	24.52 × 10^4^ to 24.87 × 10^4^	30.56 × 10^4^ to 30.83 × 10^4^
Relative standard deviation of the slope (%)	0.36	1.80	1.39	0.47	0.46	1.74	1.88	1.17
Intercept (*a*)	−7.54 × 10^3^	−1.55 × 10^4^	8.64 × 10^4^	9.28 × 10^3^	5.70 × 10^3^	1.03 × 10^4^	7.07 × 10^4^	−9.36 × 10^3^
Standard deviation of the intercept (*s*^a^)	3.25 × 10^4^	7.39 × 10^4^	1.12 × 10^5^	1.69 × 10^4^	1.52 × 10^4^	8.9010^4^	2.16 × 10^5^	1.67 × 10^5^
Confidence limit of the intercept	−7.66 × 10^3^ to −7.41 × 10^3^	−18.29 × 10^3^ to −12.70 × 10^3^	85.97 × 10^3^ to 88.82 × 10^3^	9.21 × 10^3^ to 9.34 × 10^3^	5.64 × 10^3^ to 5.75 × 10^3^	9.58 × 10^3^ to 11.02 × 10^3^	69.88 × 10^3^ to 71.51 × 10^3^	−9.42 × 10^3^ to −9.29 × 10^3^

a *y* = *a* + *bC*, where *C* is the concentration in μg mL^−1^ or ng mL^−1^ and *y* is the peak area.

b 95% confidence limit.

#### 3.2.2. Precision and accuracy

**Fig. 4:**
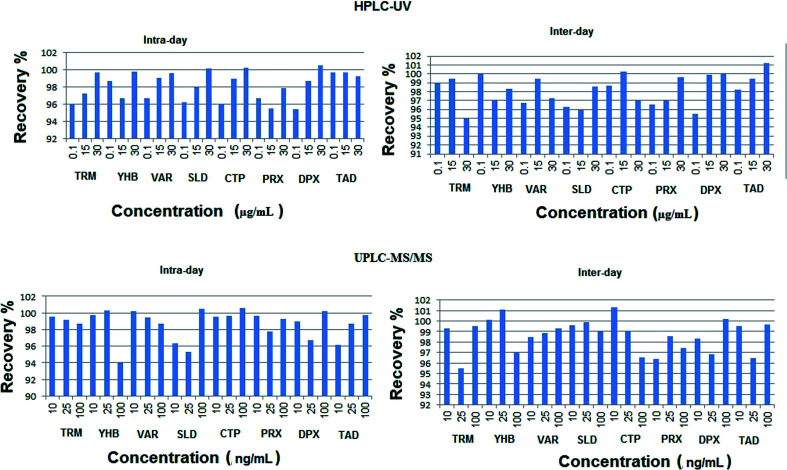
Inter-day and intra-day percentage recoveries of the studied drugs using the proposed HPLC-UV and UPLC-MS/MS methods.

The intra-day precision and also accuracy were determined by analyzing three concentration levels of working solutions of each compound on the same day (each concentration was repeated three times). Inter-day precision and accuracy have been assessed by analyzing of the three concentration levels of working solutions on three successive days. The percentage recoveries were calculated as (practical concentration/theoretical concentration) × 100, and the results are shown in Fig. 4. The concept of acceptability of the data included accuracy stated as relative error (RE %) and precision stated as relative standard deviation (RSD %). Both results of intra-day and inter-day precision and accuracy are summarized in Tables S1 and S2.†

The calculated relative standard deviation of different measurements was below 2% for UPLC-MS/MS and 2.5% for HPLC-UV, which indicates the excellent precision of the proposed analytical methods at both levels of repeatability and intermediate precision.

#### 3.2.3. Detection and quantitation limits

By following the ICH guidelines,^36^ detection and quantitation limits were determined based on the standard deviation of the response and the slope of the calibration curve using the following formulas: LOD = 3.3 × *s*/S, where *S* is the slope of the calibration curve and *s* is the standard deviation of the response; LOQ = 10 × *s*/*S*, and their theoretical values were typically evaluated in practice (Table 2).

#### 3.2.4. Selectivity

The selectivity of the methods was achieved by simply making five mixtures of the studied compounds at different concentrations within the linearity range. The mixtures were analyzed according to the preceding procedures described. Acceptable recoveries were achieved (Table S3†), revealing the high selectivity of the methods proposed to simultaneously analyze TRM, YHB, VAR, SLD, CTP, PRX, DPX and TAD.

### 3.3. Sample analysis

Fifty commercial products marketed as aphrodisiacs and suspected to contain PDE5-inhibitors were analyzed (3 honey sachets, 3 instant coffee, 16 pharmaceutical products labelled to contain natural ingredients, 18 pharmaceutical products labelled to contain SLD, 5 pharmaceutical products labelled to contain TAD, 2 pharmaceutical products labelled to contain VAR, 1 pharmaceutical product that had no content on the label, 1 pharmaceutical product labelled to contain shark extract, 1 pharmaceutical product labelled to contain SLD and DPX and the last sample labelled to contain SLD and TRM). The description, name and ingredients declared on the label of analyzed samples are listed in Table 3. The physical examination of samples showed that samples 5, 6, 15 and 44 in Table 3 were labelled to contain higher concentrations of active ingredients than the average weight of each sample, most of the samples having substandard packaging and printing. Fig. S1† presents some remarks on the packaging that shows a strong and clear indication that these products are counterfeits, sample 37 and 38 having the same name and the same lot number, different expiry dates, different words on the cap and different label colors (Fig. S1a†). Sample 33 the word “distributed” was written as “distribted” (Fig. S1b†). In sample 28, the concentration of the active ingredient was written as “135 mL” instead of “135 mg” (Fig. S1c†). Moreover, Fig. S1d† shows artifacts (cracks, faint imprints) of different tablets in samples 18, 22, 24 and 26.

**Table:** Analysis of 50 aphrodisiac products in Egypt by the proposed HPLC-UV and UPLC-MS/MS analytical methods

No.	Name	Description	Content on the label	Found by HPLC	Found by UPLC-MS/MS
1	Cialis	Blue tab	Natural	SLD 103.14 mg	SLD 103.17 mg
2	Enjoy	Green cap	Natural	SLD 52.46 mg	SLD 51.70 mg
3	Erectopril	Red cap	Natural	VAR 13.94 mg, DPX 14.34 mg	VAR 13.64 mg, DPX 14.19 mg
4	GreenValley	Green leaf shape tab	Natural	SLD 46.34 mg	SLD 46.95 mg
5	Hercules	Yellow cap and white cap	Natural 2000 mg	SLD 114.89 mg, none	SLD 114.99 mg, none
6	Man's Magic	Yellow kidney shape tab	Natural 3800 mg	SLD 100.92 mg, none	SLD 100.75 mg, none
7	MAXMAN	Golden capsule	Natural	SLD 95.64 mg, CTP 19.42 mg	SLD 95.99 mg, CTP 19.80 mg
8	MAXMAN	Black cap and green cap	Natural	SLD 115.52 mg, none	SLD 115.60 mg, none
9	Plant Viagra	Green leaf shape tab	Natural	SLD 49.04 mg	SLD 49.00 mg
10	Plant Viagra	Green leaf shape tab	Natural YHB, no conc.	SLD 51.34 mg	SLD 51.39 mg
11	Super man	Yellow cap, oily cap	Natural	SLD 100.56 mg, none	SLD 100.32 mg, none
12	Tiger king	Black tab	Natural	SLD 76.23 mg	SLD 76.33 mg
13	Top man	Black cap and white cap	Natural	SLD 89.78 mg, none	SLD 89.85 mg, none
14	Vigour 300	Blue tab	Natural 300 mg	SLD 94.87 mg	SLD 94.99 mg
15	Vigour 6800	Blue tab	Natural 6800 mg	SLD 84.43 mg	SLD 84.40 mg
16	Vigrex	White cap	Natural	SLD 61.67 mg	SLD 61.79 mg
17	Cajo-150	Yellow kidney shape tab	SLD 150 mg	SLD 113.04 mg	SLD 113.10 mg
18	Cobra-125	Red tab	SLD 125 mg	SLD 96.52 mg	SLD 96.53 mg
19	DEER-Fox	Red tab	SLD 120 mg	SLD 97.90 mg	SLD 97.94 mg
20	Erecta Power	Red cap	SLD 140 mg	SLD 96.56 mg	SLD 96.58 mg
21	Ferrari	Red tab	SLD 130 mg	SLD 127.34 mg	SLD 127.39 mg
22	FOX	Red tab	SLD 125 mg	SLD 119.43 mg	SLD 119.45 mg
23	FOX 125	Red tab	SLD 100 mg	SLD 93.56 mg	SLD 93.57 mg
24	Goldviagra	Yellow kidney shape tab	SLD 130 mg	SLD 104.12 mg	SLD 104.18 mg
25	Hard-on	Black tab	SLD 130 mg	SLD 132.46 mg	SLD 132.31 mg
26	Jaguar 120	Blue tab	SLD 120 mg	SLD 116.78 mg	SLD 116.80 mg
27	Jaguar 120	Red tab	SLD 120 mg	SLD 99.23 mg	SLD 99.31 mg
28	Plant VIGRA	Green leaf shape tab	SLD 130 mg	SLD 48.12 mg	SLD 48.20 mg
29	PureGrey	Red tab	SLD 100 mg	SLD 98.40 mg	SLD 98.51 mg
30	Vega	Blue tab	SLD 50 mg	SLD 49.81 mg	SLD 49.90 mg
31	Vega b100	Red tab	SLD 100 mg	SLD 101.54 mg	SLD 101.57 mg
32	Viag 120	Red tab	SLD 120 mg	SLD 123.42 mg	SLD 123.46 mg
33	Viagra 100	Blue tab	SLD 100 mg	SLD 87.67 mg	SLD 87.79 mg
34	Fox-125	Red tab	SLD 125, DPX 20 mg	SLD 122.78 mg	SLD 122.80 mg
35	Vega b DOL	Red tab	SLD100 mg, TRM 50 mg	SLD 96.47 mg	SLD 96.49 mg
36	Jaguar Speed	White oblong shape tab	TAD 20 mg	SLD 98.23 mg	SLD 98.20 mg
37	Cialis	Yellow tab and black cap	TAD 20 mg	None, none	None, none
38	Cialis	Yellow egg shape tab	TAD 20 mg	None	None
39	Cialis	Yellow oblong shape tab	TAD 20 mg	None	None
40	Cialis	Yellow egg shape tab	TAD 20 mg	TAD 18.85 mg	TAD 18.87 mg
41	Levitra	Yellow tab	VAR 10 mg	VAR 11.4 mg	VAR 11.5 mg
42	Levitra	Yellow tab	VAR 20 mg	None	None
43	SuperPower	Black tab	Not labelled	SLD 107.14 mg	SLD 107.15 mg
44	SHARK Extract	Oblong red tab	Shark extract 3800 mg	SLD 137.33 mg	SLD 137.45 mg
45	MAXMAN	Coffee sachets	Natural	TAD 16.84 mg	TAD 16.80 mg
46	Vitamax Power	Coffee sachets	Natural	None	None
47	Magic coffee	Coffee sachets	Natural	None	None
48	Vega Honey	Honey sachets	Natural	SLD 98.32 mg	SLD 98.39 mg
49	Royal Honey	Honey sachets	Natural	SLD 89.50 mg	SLD 89.57 mg
50	Hard-on	Honey sachets	SLD 130 mg	SLD 94.63 mg	SLD 94.65 mg

The HPLC-UV analysis revealed that 14 samples labelled to contain natural or herbal ingredients were found to contain SLD (Fig. S2a†); sample 3 was found to contain VAR and DPX (Fig. S2b†), and sample 7 was found to contain SLD and CTP (Fig. S2c†). Instant coffee sample 45 was found to contain TAD without being declared on the package (Fig. S3a†). Coffee samples 46 and 47 were found to be free from any of the studied drugs. Sample 50 that was labelled to contain honey and SLD 130 mg was found to contain SLD as labelled but at a lower concentration (94.63 mg) (Fig. S3b†). Honey samples (No. 48 and 49) were found both with and without SLD, as declared on their packages. Sample 34 was labelled to contain SLD and DPX but was found to contain only SLD; sample 35 that was labelled to contain SLD and TRM was also found to contain only SLD. Sample 36 was found to contain SLD instead of TAD. Samples 37, 38 and 39 were found to be free from any of the studied compounds although they were labelled to contain TAD. For sample 43, the constituents were not declared, and for sample 44, which was labelled to contain shark extract, was found to contain SLD. The proposed UPLC-MS/MS method was used to analyze all samples for more confirmation, and the results are listed in Table 3. The chromatograms and mass spectra of samples 3, 7, 45, and 50 are illustrated in Fig. S4–S7† as an example.

The analysis showed that most of the samples contained concentrations of active substances less than the concentrations declared on their packaging, and these concentrations remained above the permissible therapeutic doses, which requires more attention. Moreover, it confirmed that almost all samples labeled to be natural were adulterated with PDE5 inhibitors without being declared on their packages, as well as advertised and sold as being natural and safe products. This undeclared constituent may really make interaction with nitrates found in some prescription drugs such as nitroglycerin and may also decrease the blood pressure to fatal levels especially for men receiving treatment for diabetes, high blood pressure, hyperlipidemia, or heart disease who often take nitrates.

## 4. Conclusion

Due to the community heritage and the desire of Egyptians to increase their offspring, the use of sexual stimulants is very frequent, making them vulnerable to commercial fraud and drug counterfeiting. Therefore, simple analytical methods are needed to analyze these drugs to avoid their harmful effects on human health. Chromatographic HPLC/UV and UPLC-MS/MS methods have been successfully developed, optimized and also validated to simultaneously analyze VAR, SLD and TAD and their possible counterfeits TRM, YHB, CTP, PRX, and DPX in different pharmaceutical dosage forms, dietary supplements and herbal products. The HPLC method is considered as a simple, accurate and fast screening tool for counterfeit samples, while the UPLC-MS/MS is considered a highly precise, sensitive confirmatory method, which could be applied successfully to commercial products and suspicious marketed brands.

## Conflicts of interest

The authors have declared no conflict of interest.

## Supplementary Material

RA-011-D0RA10324A-s001
