# Supplementary material for: HPLC-UV and UPLC-MS/MS methods for the simultaneous analysis of sildenafil, vardenafil, and tadalafil and their counterfeits dapoxetine, paroxetine, citalopram, tramadol, and yohimbine in aphrodisiac products
Source: RSC Adv. 2021 Feb 18;11(14):8055–64. doi: 10.1039/d0ra10324a (PMC8695090; doi:10.1039/d0ra10324a)
Supplement: RA-011-D0RA10324A-s001 [file RA-011-D0RA10324A-s001.pdf]

Electronic Supplementary Material (ESI) for RSC Advances.
This journal is © The Royal Society of Chemistry 2021

Table S1: Inter-day and intra-day precision and accuracy of the proposed HPLC-UV method for TRM, YHB, VAR, SLD, CTP, PRX, DPX and TAD.

Concentration (μgmL^{-1})		Intra-day			Inter-day		
		Recovery[a]% ± *s*	RSD	RE%	Recovery[a]% ± *s*	RSD	RE%
**TRM**	0.1	96.00 ± 1.00	1.04	4.00	99.00 ± 1.73	1.75	1.00
	15	97.17 ± 1.76	1.81	2.80	99.50 ±1.32	1.33	0.50
	30	99.67 ± 1.15	1.16	0.30	95.00 ± 2.31	2.43	5.00
**YHB**	0.1	98.67± 0.58	0.59	1.30	100.00 ± 0.76	0.76	0.10
	15	96.67± 0.58	0.60	3.30	97.10 ± 1.73	1.79	2.90
	30	99.70± 0.10	0.10	0.30	98.30 ± 2.00	2.03	1.70
**VAR**	0.1	96.67 ± 1.53	1.58	3.30	96.73 ± 1.30	1.35	3.30
	15	99.00 ± 1.00	1.01	1.00	99.47 ±1.16	1.17	0.50
	30	99.57 ± 1.25	1.26	0.40	97.30 ±1.18	1.21	2.70
**SLD**	0.1	96.17 ± 1.26	1.31	3.80	96.27 ± 1.86	1.93	3.70
	15	97.90 ± 1.85	1.89	2.10	95.90 ± 1.50	1.57	4.10
	30	100.13 ± 0.70	0.70	0.10	98.57 ± 1.40	1.42	1.40
**CTP**	0.1	96.00 ± 1.00	1.04	4.00	98.67 ± 1.30	1.32	1.30
	15	98.93 ± 0.90	0.91	1.10	100.23 ± 1.22	1.22	0.20
	30	100.23 ± 0.68	0.68	0.20	97.00 ± 2.37	2.44	3.00
**PRX**	0.1	96.67 ± 1.76	1.82	3.30	96.57 ± 1.50	1.56	3.40
	15	95.50 ± 0.50	0.52	4.50	97.00 ± 1.73	1.79	3.00
	30	97.83 ± 0.76	0.78	2.20	99.67 ± 0.86	0.87	0.30
**DPX**	0.1	95.33 ± 0.58	0.61	4.70	95.53 ± 1.29	1.35	4.50
	15	98.67 ± 0.31	0.31	1.30	99.93 ± 1.01	1.01	0.10
	30	100.50 ± 0.44	0.43	0.50	100.10 ± 1.71	1.70	0.10
**TAD**	0.1	99.67 ± 1.46	1.46	0.30	98.23 2.14	2.17	1.80
	15	99.67 ± 1.15	1.16	0.30	99.50 ± 0.70	0.70	0.50
	30	99.17 ± 0.76	0.77	0.80	101.20 ± 0.72	0.71	1.20

s: standard deviation, RSD: relative standard deviation, RE : relative error
[a] Mean of three replicate measurement

Table S2: Inter-day and intra-day precision and accuracy of the proposed UPLC-MS/MS method for TRM, YHB, VAR, SLD, CTP, PRX, DPX and TAD.

Concentration ($ng\ mL^{-1}$)		Intra-day			Inter-day		
		Recovery[a]% ± *s*	RSD	RE%	Recovery[a]% ± *s*	RSD	RE%
TRM	10	99.57 ± 1.00	1.01	0.40	99.27 ± 1.24	1.25	0.70
	25	99.17 ± 0.67	0.67	0.80	95.50 ± 1.32	1.39	4.50
	100	98.67 ± 1.56	1.58	1.30	99.50 ± 1.45	1.46	0.50
YHB	10	99.73 ± 0.06	0.06	0.30	100.13 ± 1.86	1.86	0.10
	25	100.30 ±0.10	0.10	0.30	101.10 ± 0.61	0.60	1.10
	100	94.00 ± 1.00	1.06	6.00	97.00 ± 1.00	1.03	3.00
VAR	10	100.17 ± 0.72	0.72	0.20	98.47 ± 1.75	1.77	1.50
	25	99.47 ± 0.60	0.61	0.50	98.83 ± 1.76	1.78	1.20
	100	98.67 ± 1.53	1.55	1.30	99.27 ± 1.10	1.11	0.70
SLD	10	96.33 ± 1.53	1.59	3.70	99.57 ± 1.78	1.79	0.40
	25	95.33 ± 1.15	1.21	4.70	99.93 ± 0.71	0.71	0.10
	100	100.50 ± 0.96	0.96	0.50	98.97 ± 1.71	1.73	1.00
CTP	10	99.50 ± 1.40	1.41	0.50	101.30 ± 0.46	0.45	1.30
	25	99.67 ± 0.12	0.12	0.30	99.10 ± 1.91	1.92	0.90
	100	100.60 ± 0.40	0.40	0.60	96.50 ± 0.50	0.52	3.50
PRX	10	99.67 ± 0.15	0.15	0.30	96.37 ± 1.52	1.57	3.60
	25	97.73 ± 0.70	0.72	2.30	98.57± 1.91	1.94	1.40
	100	99.23 ± 1.10	1.11	0.80	97.40 ± 1.64	1.68	2.60
DPX	10	99.00 ± 1.39	1.40	1.00	98.33 ± 1.53	1.55	1.70
	25	96.73 ± 0.75	0.78	3.30	96.83 ± 0.29	0.30	3.20
	100	100.23 ± 0.59	0.58	0.20	100.17 ± 0.76	0.76	0.20
TAD	10	96.13 ± 1.40	1.46	3.90	99.50 ± 1.80	1.81	0.50
	25	98.70 ± 1.92	1.94	1.30	96.47 ± 1.50	1.56	3.50
	100	99.70 ± 0.72	0.72	0.30	99.67 ± 1.53	1.53	0.30

s: standard deviation, RSD: relative standard deviation, RE : relative error

[a] Mean of three replicate measurement.

Table S3: Determination of TRM, YHB, VAR, SLD, CTP, PRX, DPX and TAD in prepared mixtures using the proposed methods.

	HPLC															
**Mix no**	**Conc. (µg mL^{-1})**								**% Recovery[a]**							
	**TRM**	**YHB**	**VAR**	**SLD**	**CTP**	**PRX**	**DPX**	**TAD**	**TRM**	**YHB**	**VAR**	**SLD**	**CTP**	**PRX**	**DPX**	**TAD**
1	0.1	5.0	30.0	20.0	30.0	5	10.0	0.1	95.60	99.80	100.10	100.60	95.00	94.00	95.90	96.70
2	5	0.1	0.5	30.0	20.0	0.1	30.0	20.0	97.70	97.20	99.90	100.80	95.80	96.70	96.00	97.50
3	15	15	15.0	15.0	15.0	15	15.0	15.0	98.50	95.00	97.00	98.70	101.30	101.60	97.80	97.60
4	20	10	30.0	10.0	0.5	10	0.1	30.0	97.90	99.30	100.50	100.80	96.80	97.80	100.80	101.60
5	30	20	10	0.1	0.1	20	30.0	5.0	96.90	94.00	100.60	100.70	99.40	95.70	98.90	100.20
								Mean	97.32	97.06	99.62	100.32	97.66	97.16	97.88	98.72
								s	1.12	2.56	1.49	0.91	2.62	2.85	2.06	2.08
	**UPLC-MS/MS**															
**Mix no**	**Conc. (ng mL^{-1})**								**% Recovery[a]**							
	**TRM**	**YHB**	**VAR**	**SLD**	**CTP**	**PRX**	**DPX**	**TAD**	**TRM**	**YHB**	**VAR**	**SLD**	**CTP**	**PRX**	**DPX**	**TAD**
1	10	100	75	20	20	10	20	75	98.70	99.50	100.60	98.50	97.60	96.80	101.40	100.80
2	20	75	100	75	75	20	10	100	101.20	98.70	100.60	99.50	98.40	97.60	99.40	98.60
3	25	40	40	40	40	40	40	40	99.30	97.60	96.90	95.30	99.80	97.30	98.50	100.50
4	75	20	20	10	10	100	75	20	97.60	98.90	99.70	98.00	100.60	97.60	96.50	96.50
5	100	10	10	100	100	75	100	10	99.00	99.50	97.80	100.60	100.50	98.50	97.60	100.50
								Mean	99.16	98.84	99.12	98.38	99.38	97.56	98.68	99.38
								s	1.31	0.78	1.69	1.99	1.33	0.62	1.86	1.83

[a] Mean of three replicate measurement

s: standard deviation.

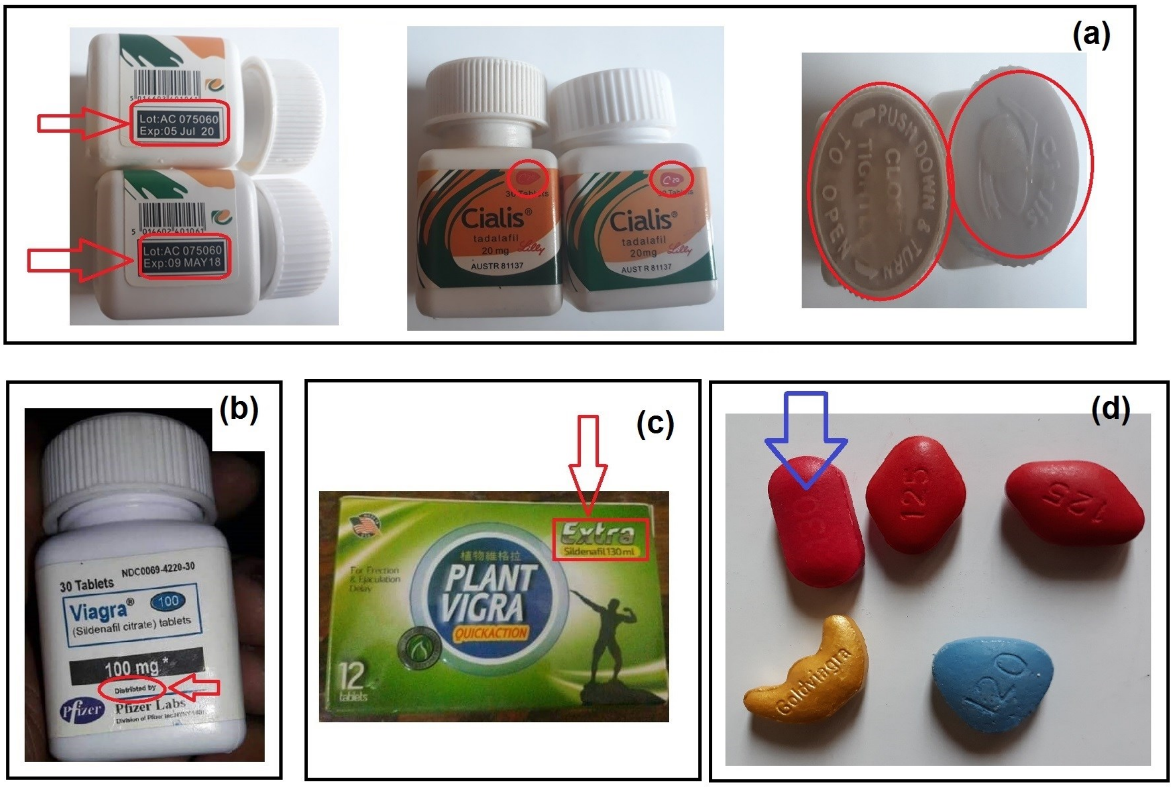

Figure S1: Different remarks indicate counterfeiting.

(a) Sample 37 and 38
(b) Sample 33
(c) Sample 28
(d) Samples 18, 22, 24 and 26

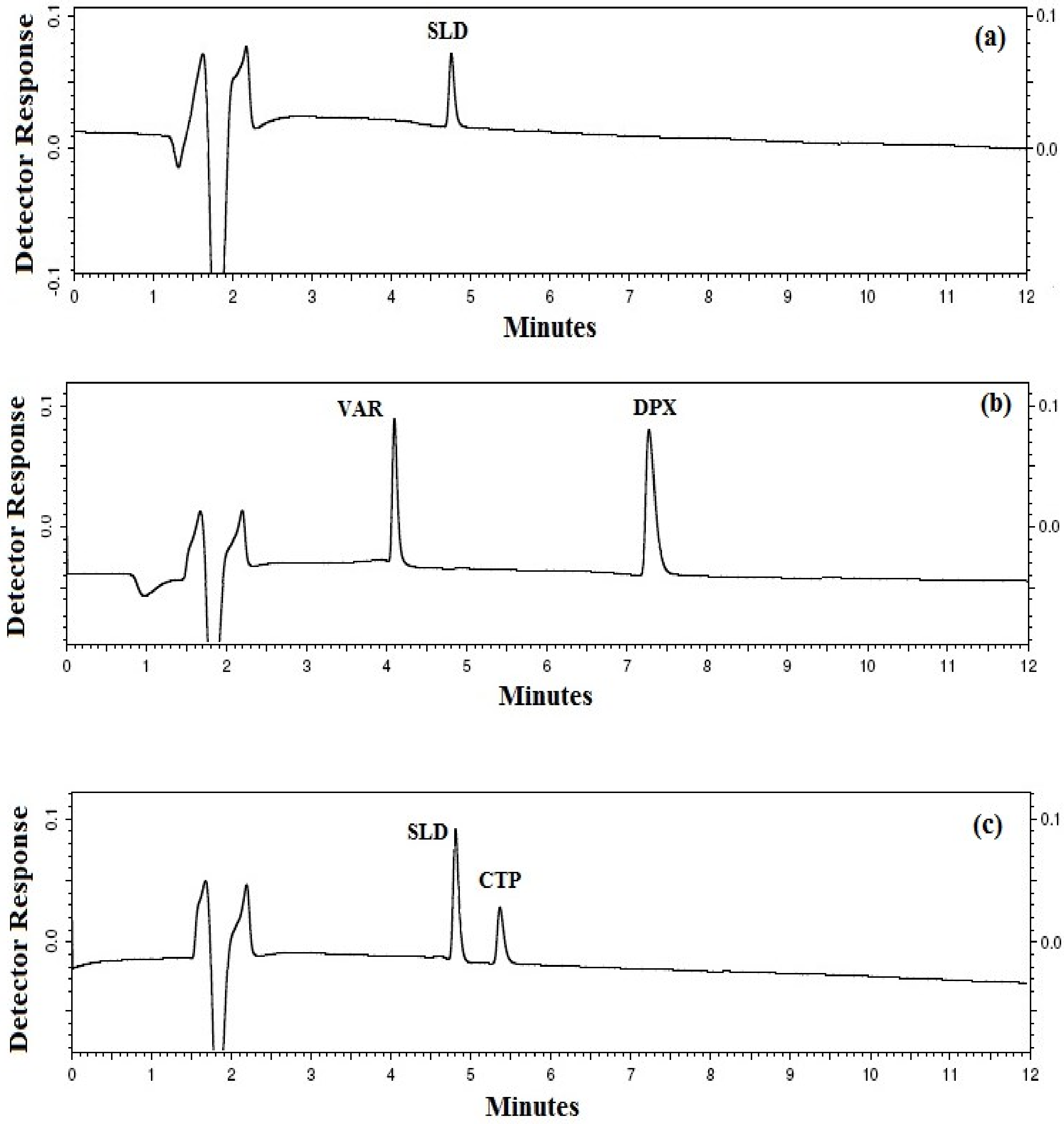

Figure S2: Typical HPLC chromatogram of (a) sample No. 4 (natural) found to contain SLD , (b) sample No. 3 (natural) found to contain VAR and DPX (c) sample No. 7 (natural) found to contain SLD and CTP.

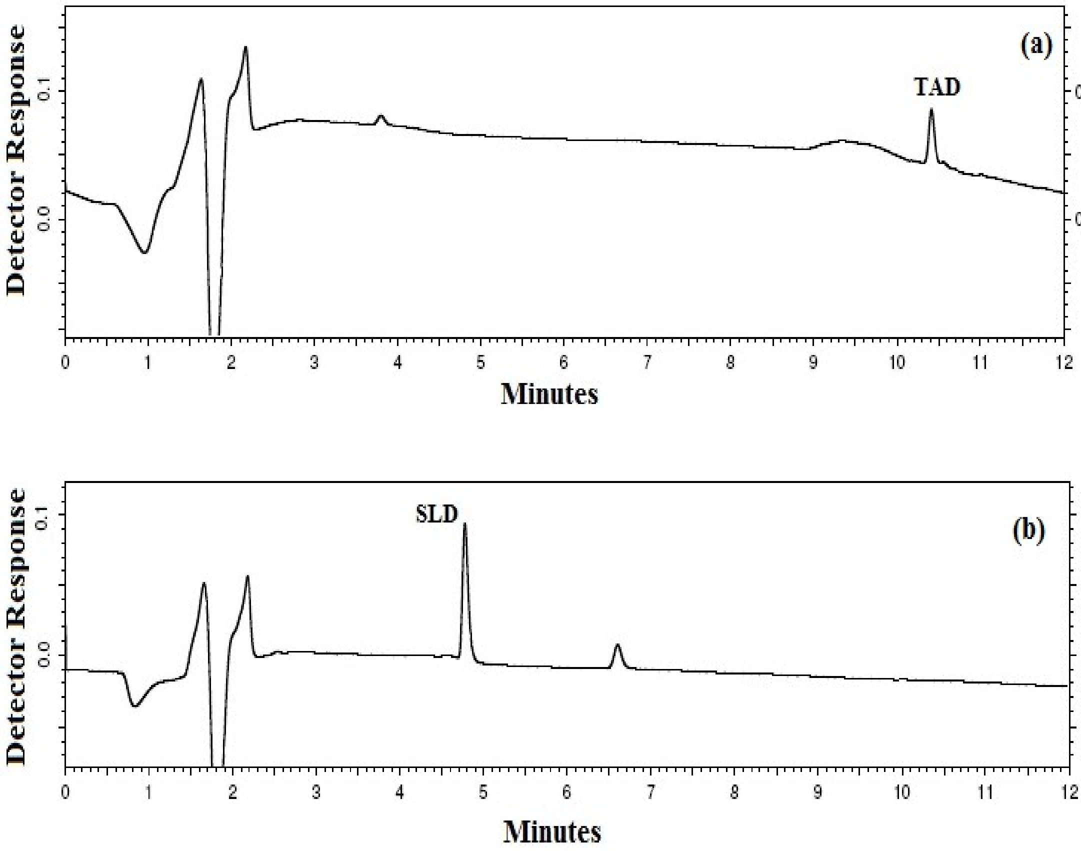

Figure S3: Typical HPLC chromatogram of (a) sample No. 45 (MAXMAN®) found to contain TAD. (b) sample No. 50 (Hard ON®) found to contain SLD.

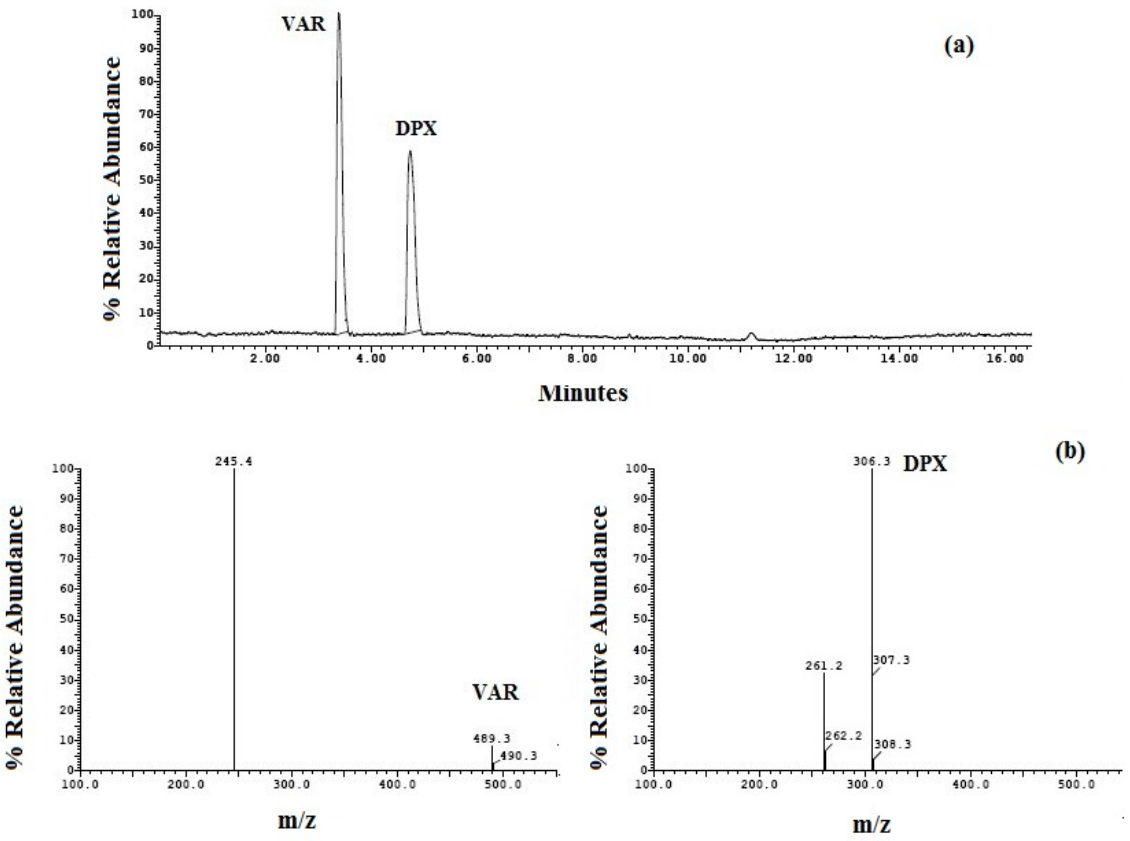

Figure S4: a) UPLC-MS/MS chromatogram of sample 3 (natural) found to contain VAR and DPX, b) Mass spectra of VAR and DPX.

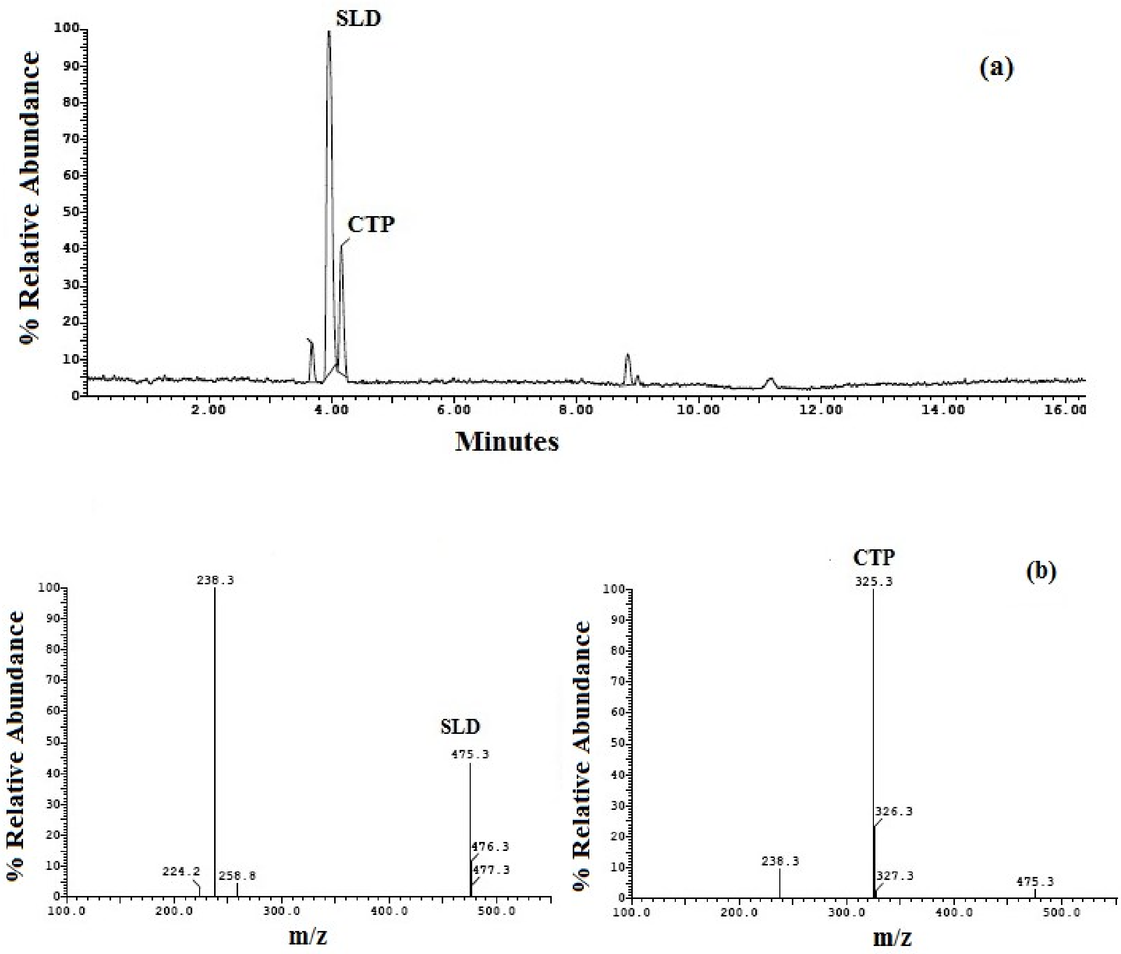

Figure S5: a) UPLC-MS/MS chromatogram of sample 7 (natural) found to contain SLD and CTP, b) Mass spectra of SLD and CTP.

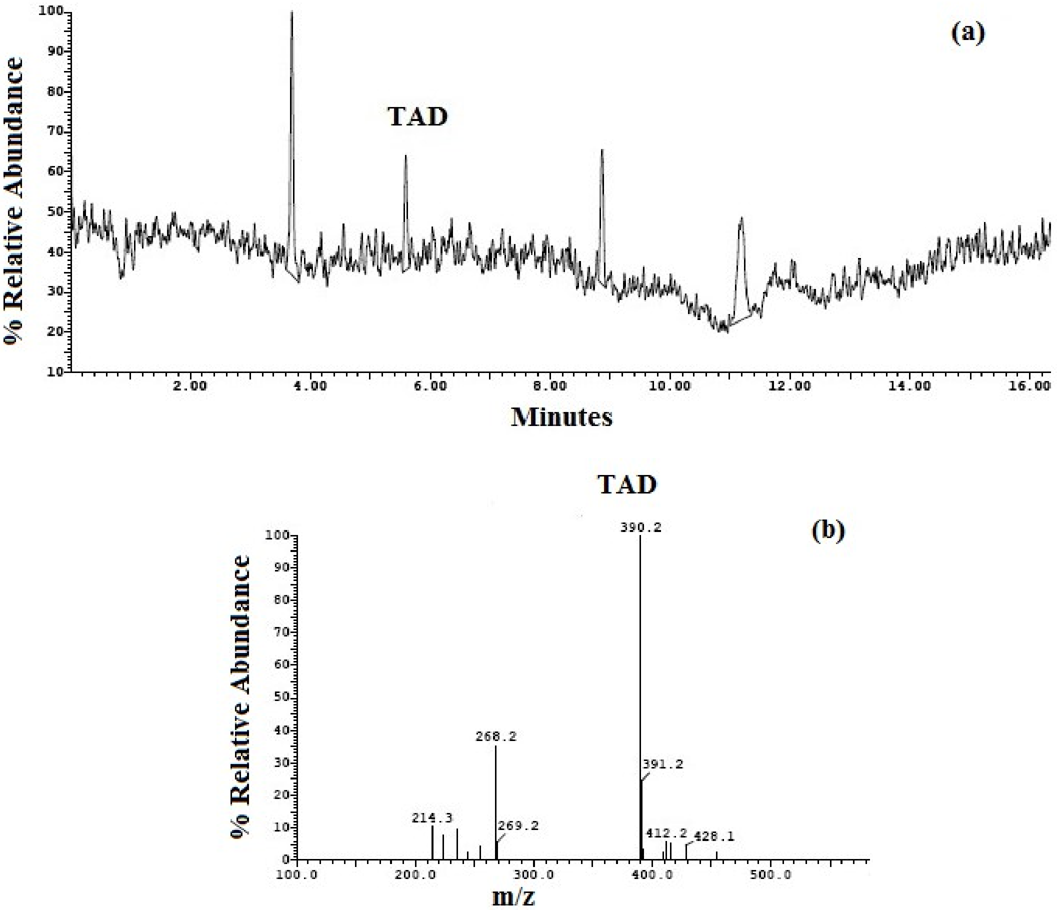

Figure S6: a) UPLC-MS/MS chromatogram of sample 45 found to contain TAD, b) Mass spectra of TAD.

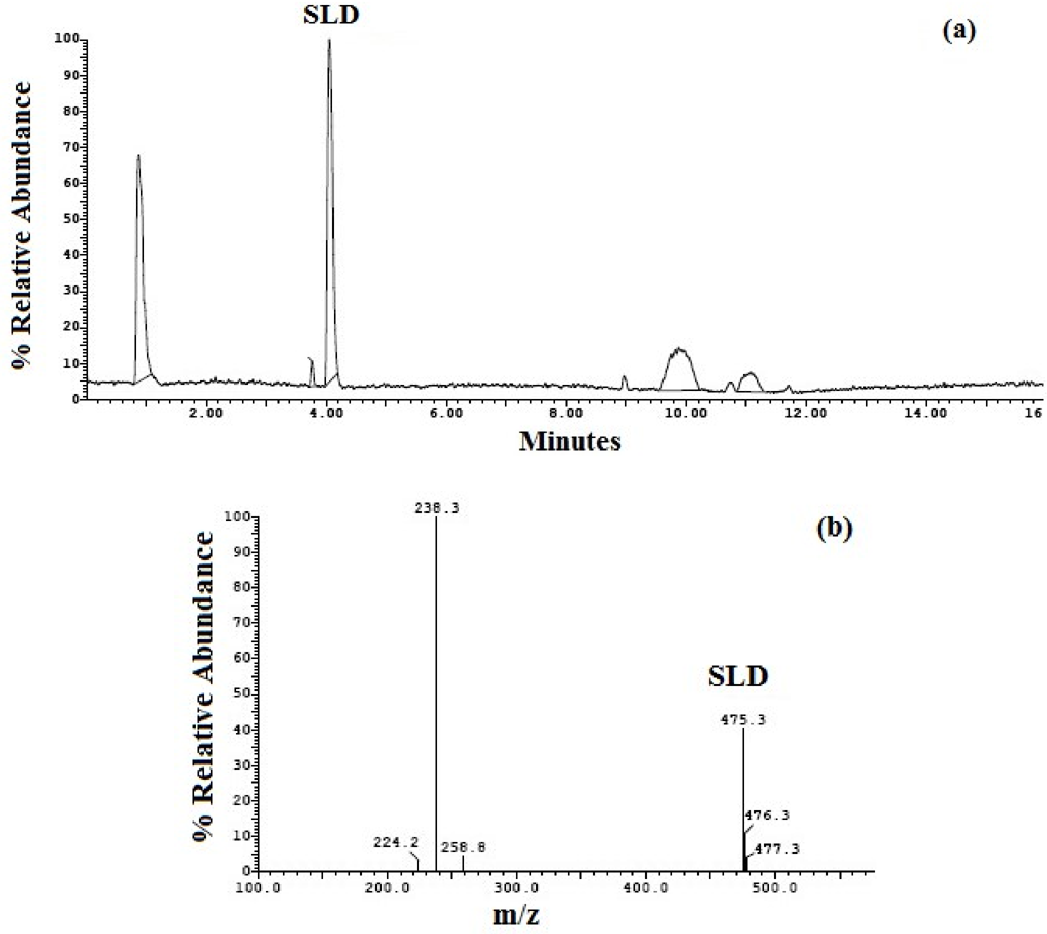

Figure S7: a) UPLC-MS/MS chromatogram of sample 50 found to contain SLD, b) Mass spectra of SLD.
